# First-in-human, Randomized, Double-blind Clinical Trial of Differentially Adjuvanted PAMVAC, A Vaccine Candidate to Prevent Pregnancy-associated Malaria

**DOI:** 10.1093/cid/ciy1140

**Published:** 2019-01-10

**Authors:** Benjamin Mordmüller, Mihály Sulyok, Diane Egger-Adam, Mafalda Resende, Willem A de Jongh, Mette H Jensen, Helle Holm Smedegaard, Sisse B Ditlev, Max Soegaard, Lars Poulsen, Charlotte Dyring, Carlos Lamsfus Calle, Annette Knoblich, Javier Ibáñez, Meral Esen, Philippe Deloron, Nicaise Ndam, Saadou Issifou, Sophie Houard, Randall F Howard, Steven G Reed, Odile Leroy, Adrian J F Luty, Thor G Theander, Peter G Kremsner, Ali Salanti, Morten A Nielsen

**Affiliations:** 1 Institut für Tropenmedizin, Universitätsklinikum Tübingen and Deutsches Zentrum für Infektionsforschung, Germany; 2 Centre de Recherches Médicales de Lambaréné, Gabon; 3 Centre for Medical Parasitology at Department of Immunology and Microbiology, University of Copenhagen and Department of Infectious Diseases, Copenhagen University Hospital; 4 ExpreS^2^ion Biotechnologies, Horsholm, Denmark; 5 Mère et Enfant face aux Infections Tropicales, Institut de Recherche pour le Développement, Université Paris 5, Sorbonne Paris Cité, France; 6 Fondation pour la Recherche Scientifique and Institut de Recherche Clinique du Bénin, Cotonou; 7 European Vaccine Initiative, Heidelberg, Germany; 8 Infectious Disease Research Institute, Seattle, Washington

**Keywords:** malaria vaccine, VAR2CSA, pregnancy-associated malaria, first-in-human, phase 1 clinical trial

## Abstract

**Background:**

Malaria in pregnancy has major impacts on mother and child health. To complement existing interventions, such as intermittent preventive treatment and use of impregnated bed nets, we developed a malaria vaccine candidate with the aim of reducing sequestration of asexual “blood-stage” parasites in the placenta, the major virulence mechanism.

**Methods:**

The vaccine candidate PAMVAC is based on a recombinant fragment of VAR2CSA, the *Plasmodium falciparum* protein responsible for binding to the placenta via chondroitin sulfate A (CSA). Healthy, adult malaria-naive volunteers were immunized with 3 intramuscular injections of 20 μg (n = 9) or 50 μg (n = 27) PAMVAC, adjuvanted with Alhydrogel or glucopyranosyl lipid adjuvant in stable emulsion (GLA-SE) or in a liposomal formulation with QS21 (GLA-LSQ). Allocation was random and double blind. The vaccine was given every 4 weeks. Volunteers were observed for 6 months following last immunization.

**Results:**

All PAMVAC formulations were safe and well tolerated. A total of 262 adverse events (AEs) occurred, 94 (10 grade 2 and 2 grade 3) at least possibly related to the vaccine. No serious AEs occurred. Distribution and severity of AEs were similar in all arms. PAMVAC was immunogenic in all participants. PAMVAC-specific antibody levels were highest with PAMVAC-GLA-SE. The antibodies inhibited binding of VAR2CSA expressing *P. falciparum*-infected erythrocytes to CSA in a standardized functional assay.

**Conclusions:**

PAMVAC formulated with Alhydrogel or GLA-based adjuvants was safe, well tolerated, and induced functionally active antibodies. Next, PAMVAC will be assessed in women before first pregnancies in an endemic area.

**Clinical Trials Registration:**

EudraCT 2015-001827-21; ClinicalTrials.gov NCT02647489.

## Teaser

The pregnancy-associated malaria-vaccine candidate PAMVAC is safe and well tolerated with all three tested formulations and induces functional binding-inhibitory antibodies. The vaccine with glucopyranosyl lipid adjuvant (GLA) in stable emulsion is superior to Alhydrogel and a GLA/QS21 liposomal formulation.

The vast majority of malaria episodes occur in sub-Saharan Africa. With continuous exposure to the main responsible parasite, *Plasmodium falciparum*, semi-immunity develops over time, such that young children, especially, are at highest risk of developing severe malaria. During pregnancy, the previously acquired immunity is no longer sufficient, and significant morbidity and mortality of the mother and the child may occur. It is estimated that pregnancy-associated malaria (PAM) results in 20 000 maternal and 200 000 infant deaths annually [1].

*Plasmodium falciparum*–infected red blood cells (iRBCs) sequester in the vasculature including the placenta [2] and thereby avoid removal in the spleen. Sequestration is mediated by members of the *P. falciparum* erythrocyte membrane protein 1 (PfEMP1) family, which are expressed on the surface of iRBCs [3]. Each parasite genome contains approximately 60 *var* genes that code highly variable PfEMP1 proteins, mediating binding to multiple different endothelial receptors [4]. Broadly reactive antibodies against PfEMP1 are acquired progressively as a result of repeated infections, protecting in concert with other immune responses first against severe forms of malaria and, thereafter, against uncomplicated infections [5–7]. One member of the PfEMP1 family, VAR2CSA, mediates binding to a distinct placenta-specific form of chondroitin sulfate A (CSA) [8]. Placental CSA is mainly attached to the proteoglycan syndecan-1 present in the maternal intervillous space and on the syncytiotrophoblast cells [9]. Thus, parasites that express VAR2CSA are only rarely found in nonpregnant hosts; hence, protective anti-VAR2CSA immunoglobulin G (IgG) only develop during pregnancy [10] when significant exposures to VAR2CSA-expressing *P. falciparum* occur [11, 12]. The level of circulating anti-VAR2CSA IgG predicts protection against PAM during successive pregnancies [13–17]. The main mechanism of protection is interference with iRBCs binding to placental CSA [10, 18]. In animal models, vaccination with recombinant subfragments of the VAR2CSA protein elicit binding inhibitory anti-VAR2CSA antibodies with characteristics similar to those acquired in pregnant women through natural exposure, findings that have motivated the development of a vaccine [14, 19–26].

The preferred product characteristic of a placental malaria vaccine is induction of lifelong protection with 1 dose given to adolescent girls. This could be given together with the human papillomavirus vaccine to adolescent girls and would have several advantages over the World Health Organization’s recommended methods that consist of intermittent treatment, bed nets, and prompt diagnosis. Long-lasting vaccines offer a much greater window of protection than any chemoprophylactic intervention. Vaccine-induced protection can be initiated before the first pregnancy, supported by bed nets at a critical time period because pregnancy could proceed unnoticed or unreported [27]. However, whether a vaccine will be able to stand alone remains to be tested.

Following extensive evaluation, the lead candidate PAMVAC was identified [20]. It spans the interdomain region 1 through Duffy binding-like domain 2 to interdomains 2 (ID1-ID2a) of VAR2CSA. Immunization of rodents with PAMVAC induces adhesion-blocking antibodies with strain-transcending activity [20]. We used Alhydrogel as comparator adjuvant due to the extensive clinical experience, availability, and well-known safety profile. To test if antibody responses could be augmented and sustained, we used 2 formulations of the Toll-like receptor 4 agonist glucopyranosyl lipid adjuvant (GLA), developed and provided by the Infectious Disease Research Institute (IDRI; Seattle, WA). The first GLA formulation was a stable squalene emulsion (GLA-SE) and the second was a liposome formulation also containing QS21/saponin (GLA-LSQ).

Here, we report results of the first-in-human phase 1 clinical trial of 20 μg or 50 μg PAMVAC, formulated with Alhydrogel, GLA-SE, or GLA-LSQ in malaria-naive volunteers in Tübingen, Germany. Importantly, the antibodies acquired were functional. The activity was influenced by formulation in particular GLA-SE were highly immunogenic.

## METHODS

### Study Design and Procedures

The study was conducted in a malaria-naive cohort in Tübingen, Germany. The study was done concurrently with a trial in Cotonou, Benin, as part of a 2-center, randomized, staggered, adjuvant-selection, dose-escalation clinical phase 1 trial. Eligible volunteers were immunized with PAMVAC, adjuvanted with Alhydrogel, GLA-SE, or GLA-LSQ 3 times at 4-week intervals. Vaccinations were given by intramuscular injection in the deltoid. Injections were given on alternating sides, starting with the nondominant arm. Neither the clinical team nor the volunteers were aware of allocation to the Alhydrogel, GLA-SE, or GLA-LSQ arm. An independent pharmacist prepared the syringes with masking tape. Vaccinations started in 3 sentinel volunteers, receiving 20 μg PAMVAC adjuvanted with Alhydrogel, GLA-SE, or GLA-LSQ, respectively, followed by 2 volunteers in each group 1 day later (n = 3 per arm). At least 4 weeks later, the second group receiving 3 times 50 μg PAMVAC was started. The same procedures were applied as in the first group. All volunteers were examined by a physician 7 and 28 days following each vaccination. In addition, volunteers were contacted actively by the clinical team 1 and 14 days following each vaccination. Long-term follow-up visits were performed 16 and 28 weeks following the last vaccination (days 168 and 252). All volunteers were encouraged to contact the clinical team in case of any adverse event (AE). During the study period, the team was available 24 hours, 7 days a week.

The primary endpoint of the trial was the number and grade of AEs and serious AEs (SAEs) possibly, likely, or definitely related to vaccination. Severity of AEs was graded as mild (grade 1), moderate (grade 2), severe (grade 3), or potentially life threatening (grade 4). For laboratory and solicited AEs, adapted Brighton Collaboration [28] and Food and Drug Association toxicity grading scales were used. The secondary endpoint was the PAMVAC-specific IgG concentration. Seroconversion was defined as reactivity above the average reactivity in plasma of 10 unexposed individuals plus 2 standard deviations.

The trial was approved by the ethics committee of the Medical Faculty and the University Clinics of the University of Tübingen and the German Regulatory authorities. It strictly adhered to International Council for Technical Requirements for Human Use guidelines and the principles of the Declaration of Helsinki.

### Vaccine

The PAMVAC vaccine candidate consists of an antigen encompassing the ID1-DBL2-ID2a subunits of the FCR3 variant of VAR2CSA [20]. The vaccine was manufactured according to current Good Manufacturing Practice (cGMP) guidelines using a conventional batch process based on a cGMP-compliant Master Cell Bank of a *Drosophila* Schneider 2-derived cell line (Expres^2^ion, Denmark). Cells were expanded in shakers and Cultibags for 3 weeks. All medium components used for the upstream process were free of animal components. After 3 days in the production bioreactor, the bulk harvest was clarified by depth filtration, and the product was purified by a multistep process including SP Sepharose chromatography, virus removal (low pH treatment), Capto Adhere chromatography, Sartobind Q membrane polishing, Planova 20 N virus removal filtration, and an ultrafiltration/diafiltration step to reach the final concentration of 0.4 mg/mL. The drug product was aseptically vialed at Miltenyi (Germany) and stored at –20°C. The bulk batch of Alhydrogel was produced at Brenntag (Denmark), diluted, and filled at Nova Laboratories (United Kingdom) under cGMP and stored at 2°C–8°C. GLA-SE and GLA-LSQ were manufactured by the IDRI and stored at 2°C–8°C. The vaccine was prepared extemporaneously and passed to the clinical team in a syringe containing either 20 μg or 50 μg PAMVAC. The 20-μg PAMVAC preparations contained 0.17 mg Alhydrogel or 1 μg GLA with either 2% squalene (GLA-SE) or 0.4 µg QS21 (GLA-LSQ); the 50-μg PAMVAC contained 0.43 mg Alhydrogel or 2.5 μg GLA with either 2% squalene or 1 µg QS21, respectively.

### Immunological Investigations

#### Antibody Quantification

PAMVAC-specific IgG was measured by indirect enzyme-linked immunosorbent assay (ELISA). Specific IgG present in the plasma bind to the ID1-ID2a coated onto the microplate wells and detected by horseradish peroxidase (HRP) conjugated goat anti-human IgG antibody. HRP activity was measured by color conversion of the substrate o-phenylenediamine dihydrochloride in the presence of hydrogen peroxide. Absorbance at 490 nm was measured after stopping the reaction with 0.5 M sulfuric acid. The optical density at different plasma dilutions was measured with a SpectraMax plate reader using SoftMax Pro GxP software. The level of PAMVAC-specific plasma IgG was defined as the area under the curve (AUC) of the sigmoid titration curve of 11 two-fold dilutions starting from 1:100. Titration curves were made for each volunteer at each day of study (day 7, 28, 35, 56, 63, 84, and 252). The binding to heterologous ID1-ID2a variants was measured using a single dilution (1:400).

#### Antibody Function

The ability of immunoglobulins to interfere with the binding of iRBC expressing native VAR2CSA was measured as previously described [29]. Briefly, parasite cultures containing *P. falciparum* ring-stage iRBC were exposed to tritium-labeled hypoxanthine for 24 hours, after that parasites were on late-stage trophozoite and schizont stages. A total of 200 000 cells per well were incubated for 2 hours at 37°C in a 96-well plate (Falcon 351172) coated the day before with 2 µg/mL CSA (D8428 Sigma-Aldrich) and blocked with 1% immunoglobulin-free bovine serum albumin (A7030 Sigma-Aldrich). Wells were run in triplicate either without plasma or with 4 two-fold dilutions from 1:10 of a pool of plasma from malaria-naive, a pool of plasma samples from malaria-exposed multiparous women from Benin [30], or test plasma in a total volume of 100 µL. Unbound iRBCs were washed away by a pipetting robot (Biomek 2000, Beckman Coulter). Subsequently, the adhering cells were harvested to a filter plate (Perkin-Elmer) and recorded by liquid scintillation counting on a Topcount NXT (Perkin-Elmer).

### Statistical Analyses

Eligible participants were randomized on the day of first immunization by an independent party that used a computer-generated allocation table with participant identification codes. Sealed envelopes were provided to the pharmacist immediately before vaccination. The study was an exploratory phase 1 clinical trial. Hence, sample size was chosen to allow detection of large differences in AE pattern and PAMVAC-specific IgG concentration; that is, with 9 participants per group, 10% vs 80% of at least possibly related AEs and a difference in normally distributed variables larger than 60% of the standard deviation could be detected with 90% power and 5% alpha. Except for predefined immunological analyses, all statistical modeling was exploratory. A 2-tailed alpha <5% was considered statistically significant. All exploratory immunological analysis included dose and allocation as variables (ie, to calculate differences between groups) if not otherwise stated. Clinical and demographic data were captured using OpenClinica, version 3.9.1; calculations were done using R, version 3.4.3.

## RESULTS

### Study Population

**Figure 1.**
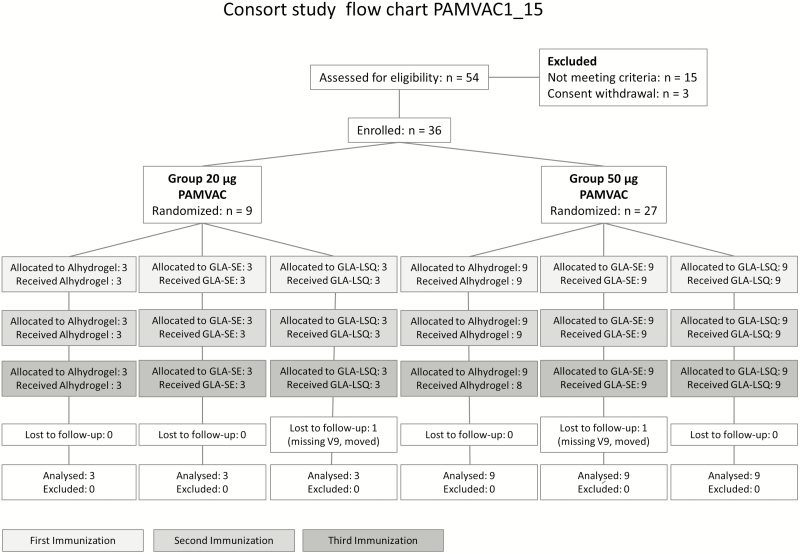
Study flow chart. Abbreviations: GLA-LSQ, glucopyranosyl lipid adjuvant containing QS21/saponin; GLA-SE, glucopyranosyl lipid adjuvant in stable emulsion; PAMVAC, pregnancy-associated malaria vaccine.

The study took place between 4 May 2016 and 9 March 2017. In total, 36 volunteers were recruited, 9 received 20 µg and 27 received 50 µg PAMVAC. Immunization was performed between 9 May 2016 and 30 August 2016. All vaccinations were given within the prespecified time window of 28–35 days. One volunteer, allocated to 50 μg PAMVAC-Alhydrogel, did not receive the third injection due to travel abroad. This volunteer remained in the study and follow-up continued until the last visit. One volunteer, allocated to PAMVAC-GLA-SE, missed the last follow-up visit (D252) because of relocation (Figure 1).

Baseline demographics were similar between the groups (Table 1), which represented young, malaria-naive adults, 5 with a body mass index >30 kg/m^2^ (30.1, 30.5, 32.7, 36.7, and 40.3 kg/m^2^) and 1 with 18.3 kg/m^2^.

**Table 1.** Baseline Demographics of the Study Volunteers

	Age, y^a^	Sex^b^	Weight, kg^a^	Body Mass Index,^a^ kg/m^2^
20/Al	23.9 (21.3; 32.3)	1:2	69 (51; 81)	22.3 (18.7; 25.6)
20/GLA-SE	27.9 (23.2; 29.3)	2:1	81 (71; 82)	28.7 (22.7; 30.5)
20/GLA-LSQ	20.4 (19.4; 23.4)	3:0	64 (62; 79)	20.9 (18.3; 28.0)
50/Al	24.2 (19.9; 35.3)	6:3	74 (53; 107)	22.8 (19.7; 40.3)
50/GLA-SE	25.3 (20.8; 33.3)	2:7	82 (59; 138)	25.3 (21.0; 36.7)
50/GLA-LSQ	22.8 (18.3; 36.4)	6:3	80 (53; 92)	25.7 (19.5; 32.7)

Abbreviations: GLA-LSQ, glucopyranosyl lipid adjuvant containing QS21/saponin; GLA-SE, glucopyranosyl lipid adjuvant in stable emulsion.

^a^Median (min; max).

^b^n, female:male.

In general, PAMVAC was well tolerated (Table 2). No SAEs but 262 AEs (188 grade 1, 62 grade 2, 10 grade 3, and 2 grade 4) occurred in 36 of the 36 volunteers, 94 at least possibly related to PAMVAC (Table 2). Both grade 4 AEs were laboratory abnormalities observed in 1 volunteer allocated to 20 μg PAMVAC-Alhydrogel who presented with elevated aspartate aminotransferase (354 U/L) and creatinine kinase (14 000 U/L) concentrations without clinical symptoms or other laboratory abnormalities. The laboratory values were normal at the next visit 17 days later and were judged unlikely related to vaccination (before the visit, the volunteer performed heavy-weight strength training after a prolonged period without exercise). Of the 10 grade 3 AEs, 2 were at least possibly related. One volunteer (20 μg PAMVAC-GLA-SE) experienced an episode of fever on the day of first injection, another (50 μg PAMVAC-GLA-LSQ) developed grade 3 injection-site swelling (diameter, approximately 10 cm) on the day of the third injection. Both grade 3 adverse reactions were transient and resolved without sequelae. AE patterns were similar between the 3 adjuvants and did not increase in frequency or severity at booster doses (Supplementary Table 1). Both dose levels (20 and 50 µg) were similarly well tolerated.

**Table 2.** Related Adverse Events

Severity	20/Al (n = 3)^a^	20/GLA-LSQ (n = 3)^a^	20/GLA-SE (n = 3)^a^	50/Al (n = 9)^a^	50/GLA-LSQ (n = 9)^a^	50/GLA-SE (n = 9)^a^
1	8 (3)	7 (2)	7 (3)	17 (8)	24 (7)	19 (7)
2	1 (1)	0	0	1 (1)	2 (1)	6 (4)
3	0	0	1 (1)	0	1 (1)	0

Abbreviations: GLA-LSQ, glucopyranosyl lipid adjuvant containing QS21/saponin; GLA-SE, glucopyranosyl lipid adjuvant in stable emulsion.

^a^Number of adverse events (number of volunteers).

### Immunogenicity

**Figure 2.**
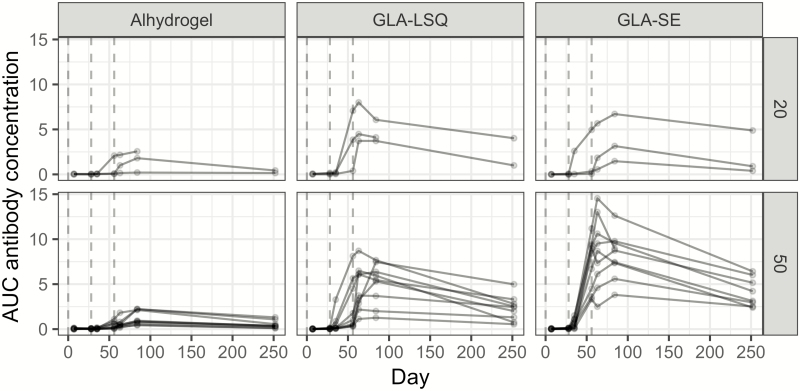
Pregnancy-associated malaria vaccine (PAMVAC) immunoglobulin G (IgG) over time. Enzyme-linked immunosorbent assay was used to measure IgG antibody reactivity generated against PAMVAC. The *y*-axis shows the area under the curve (AUC) calculated for each individual at day 7, 28, 35, 56, 63, 84, and 252. The AUC unit was derived from recording the optical density values in plasma after 11 two-fold dilutions (from 1/100 to 1/102 400), yielding a titration curve under which the area was calculated. Shown are the AUC values for individuals who received the 20-µg dose (upper panel) and 50-µg dose (lower panel) according to adjuvant and days after first vaccination. Each individual was vaccinated at time 0, 28, and 56 days (vertical dotted lines). Abbreviations: GLA-LSQ, glucopyranosyl lipid adjuvant containing QS21/saponin; GLA-SE, glucopyranosyl lipid adjuvant in stable emulsion.

PAMVAC induced seroconversion in all volunteers irrespective of dose level and adjuvant (Figure 2). However, a significant difference in the median AUC IgG measurements was observed for volunteers allocated to receive PAMVAC formulated with GLA-based adjuvants compared to Alhydrogel. Relative to the Alhydrogel group, PAMVAC-IgG plasma levels 4 weeks after the last vaccination were highest in the GLA-SE group (6.2-fold higher; 95% confidence interval [CI], 3.6–10.7), followed by the GLA-LSQ group (4.4-fold higher; 95% CI, 2.6–7.6). This difference appeared more pronounced at the end of follow-up (day 252) with 8.5-fold (95% CI, 4.3–16.6) and 5.5 fold (95% CI, 2.8–10.8) increases in the GLA-SE and GLA-LSQ compared to the Alhydrogel group, respectively. However, in all groups, the plasma levels decreased from day 84 to day 252 with no significant effect of allocation and dose.

Time to peak PAMVAC-IgG concentration was shorter in volunteers vaccinated with PAMVAC-GLA-SE (9 days earlier; 95% CI, 1–17) and PAMVAC-GLA-LSQ (12 days earlier; 95% CI, 4–20) compared to PAMVAC-Alhydrogel. A comparison of peak concentrations instead of total PAMVAC-IgG at 4 weeks following last immunization (day 84) showed a similar fold difference in concentration of GLA-based compared to Alhydrogel-based adjuvants.

**Figure 3.**
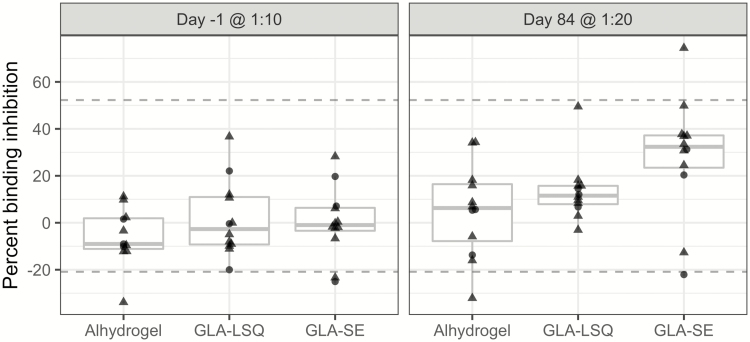
Functional activity of induced antibodies. The binding inhibitory activity of plasma against erythrocytes infected with VAR2CSA expressing FCR3 parasites binding to chondroitin sulfate A (CSA). Samples from individuals enrolled in the study, a pool of plasma from naive Danes, and a pool of plasma from multiparous women living in a highly endemic area in Benin. The plasma was tested at day -1 at a dilution of 1:10 and at day 84 at a 1:20 dilution. Briefly, CSA was coated at the bottom of 96 well plates. Infected erythrocytes were added along with plasma. The plates were washed using a washing robot (Biomek 2000). Values shown are the percentage inhibition according to binding in wells without plasma after subtraction of background binding values in wells without CSA. The upper and lower horizontal lines indicate the positive and negative control plasma samples. All samples were run in triplicate. The 50-µg group is indicated by triangles; 20-µg group is indicated by circles. See also Supplementary Figure S2. Abbreviations: GLA-LSQ, glucopyranosyl lipid adjuvant containing QS21/saponin; GLA-SE, glucopyranosyl lipid adjuvant in stable emulsion.

The functional response that protects against placental malaria may include many effector mechanisms. However, an antibody response that hinders iRBC sequestering, thereby alleviating detrimental downstream inflammation events that take place in the placenta, appears the most plausible and well-documented mechanism of protection [31–33]. Our binding inhibition assay measures the ability of in vitro cultured VAR2CSA-expressing iRBC to bind to the placental receptor CSA coated to a 96-well plate in the presence of plasma IgG [29, 34]. Four weeks following the last immunization, binding inhibition at a 1:20 dilution of plasma was significantly higher in volunteers vaccinated with GLA-SE (23% difference to Alhydrogel; 95% CI, 7–40) compared to GLA-LSQ and Alhydrogel formulated PAMVAC (Figure 3). This difference was not significant at 1:10 dilution (Supplementary Figure S1). The overall level of antibodies against PAMVAC measured by ELISA was correlated linearly with the level of binding inhibition at 1:20 dilution (*P* = .0005; Supplementary Figure S2). In contrast, the level of functional antibodies was significantly higher in mice when the PAMVAC antigen was formulated with GLA-LSQ than in GLA-SE or Alhydrogel (Supplementary Figure S3).

**Figure 4.**
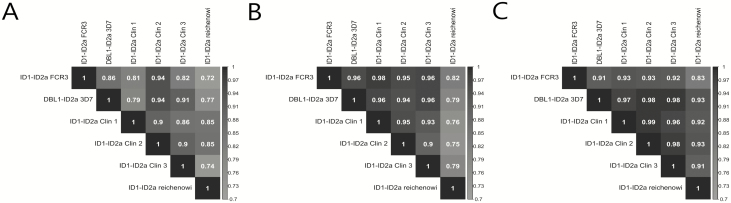
Correlation matrix of immunoglobulin G (IgG) reactivity between VAR2CSA variants. Alhydrogel (*A*), glucopyranosyl lipid adjuvant containing QS21/saponin (*B*), and glucopyranosyl lipid adjuvant in stable emulsion (GLA-SE) (*C*). The shading is proportional to the level of cross-reactivity measured by correlating the reactivity of IgG in plasma from individuals in each arm of the study. Numbers indicated according to the shading are r^2^-values, denoting how close the data are to the fitted regression line. Note the overall higher correlation in the GLA-SE adjuvanted group. See also Supplementary Figure S4.

Due to substantial sequence variability even in the minimal binding domain of VAR2CSA, we investigated the level of cross-reactivity of PAMVAC-induced antibodies on day 84 by ELISA. We found that immunized volunteers acquired substantial levels of antibodies with specificity for the PAMVAC region of genetic variants representing different clades of VAR2CSA (Figure 4). The highest level of correlation was in PAMVAC-GLA-SE vaccinated volunteers with a pattern that represented genetic distance (ie, the *Plasmodium reichenowi* variant being least similar to the vaccine antigen; Supplementary Figure 4).

## DISCUSSION

To our knowledge, this is the first report of a clinical trial testing a vaccine to generate an antibody response against the molecular determinants of a virulence factor of *P. falciparum* malaria. The vaccine was well tolerated with only 2 transient and at least possibly related grade 3 AEs. One volunteer experienced an episode of fever on the day of first injection, and another developed an injection-site swelling. In contrast to preclinical findings in rodents and rabbits demonstrating induction of high levels of antibodies using GLA-LSQ compared to Alhydrogel or GLA-SE (see Supplementary Figure 4), we found that PAMVAC formulated with GLA-LSQ did not improve immunogenicity compared to GLA-SE. Peak response and time to peak were superior to the Alhydrogel formulation with both GLA-based formulations. Interestingly, the plasma of GLA-SE–vaccinated volunteers had the highest binding-inhibitory capacity. We observed substantial binding of IgG to heterologous PAMVAC recombinant proteins, even as diverse as the VAR2CSA variant from the *P. reichenowi* genome. Again, the GLA-SE formulation showed the highest cross-reactivity.

Currently, there is no valid surrogate measure for protection against placental malaria [35]. Validation of such a surrogate will need a large trial powered to measure efficacy. Nevertheless, naturally acquired protection is correlated with both high levels of IgG against VAR2CSA per se and IgG that targets epitopes involved in binding to CSA and therefore qualify as plausible candidates. Is it possible to assess whether antibody responses such as those acquired during first pregnancy by women living in areas of high malaria endemicity are functionally equivalent to vaccine-induced ones? Antibodies acquired during first pregnancy may result from repeated infections during pregnancy or even a single mosquito inoculating several different parasites. This complexity impedes the identification of structural features or feature patterns that define a protective response, as naturally acquired antibodies may be both redundant and targeting nonprotecting epitopes. The PAMVAC antigen can induce cross-reactive binding-inhibitory antibodies in rodents at a level similar to that found in hyperimmune women [20]. In German malaria-naive individuals, it appears that vaccine-induced IgG targets both conserved epitopes and epitopes involved with specific binding to CSA, but at a lower level than observed during both preclinical investigations and in multiparous women living in endemic areas. However, it is not clear how this property translates into protective efficacy of a VAR2CSA-based vaccine.

Previous phase 1 studies with malaria vaccines have shown that immune responses can be significantly lower in endemic populations, but it is also conceivable that vaccine responses may be augmented by previous exposure [36, 37]. These questions will be addressed in measurements of IgG longevity and cross-reactivity of PAMVAC-vaccinated nulligravid women in Benin. Also, studies on the specific amino acids targeted are ongoing to elucidate whether structural modifications can be made in order to generate a more potent second-generation PAMVAC capsid-like particle vaccine [38, 39].

In conclusion, PAMVAC formulated with Alhydrogel, GLA-SE, or GLA-LSQ is well tolerated in healthy, malaria-naive adults. The antigen induces a functional IgG response and is most immunogenic when formulated with GLA-SE.

## Supplementary Data

Supplementary materials are available at Clinical Infectious Diseases online. Consisting of data provided by the authors to benefit the reader, the posted materials are not copyedited and are the sole responsibility of the authors, so questions or comments should be addressed to the corresponding author.

ciy1140_suppl_Supplementary_Figure_S1

ciy1140_suppl_Supplementary_Figure_S2

ciy1140_suppl_Supplementary_Figure_S3

ciy1140_suppl_Supplementary_Figure_S4

ciy1140_suppl_Supplementary_Table_1

ciy1140_suppl_Supplementary_Figure_Legends

## Notes

***Acknowledgments.*** The authors thank the team of the Clinical Trial Platform at the Institute of Tropical Medicine of the University of Tübingen and the German Center for Infection Research for generous support and help. They thank Anne Corfitz for technical assistance with parasite cultures and binding assays; Bezim Berisha and Elham Alijazaeri for technical assistance with protein expression and purification; Nahla Chehabi for technical assistance with DNA cloning; and Nicola Stingelin for ethical reviews.

***Financial support.*** This work was supported by the European Union in the Seventh Framework Programme (FP7-HEALTH-2012-INNOVATION; under grant agreement 304815), the Danish Advanced Technology Foundation (under grant number 005-2011-1), and a Medium Scale Collaborative Project supported by the German Federal Ministry of Education and Research (Bundesministerium für Bildung und Forschung) through EVI, KfW, and Irish Aid. This work was supported in part by the Bill and Melinda Gates Foundation (grant numbers 42387 and OPP1055855 to S. G. R.).

***Potential conflicts of interest.*** W. A. d. J. and C. D. have a patent issued on the S2 Expression platform (PCT/EP2009/057278). L. P., W. A. J., C. D., and M. aS. were employed by ExpreS^2^ion Biotechnologies during the study. T. G. T. and A. S. have a patent issued on VAR2CSA vaccines (PCT/DK2003/000938). T. G. T., A. S., and M. A. N. appear on a patent issued on virus like particle vaccines (US10086056B2) and are co-owners and founders of Nextgen Vaccines and AdaptVac that develops VLP vaccines. M. E. reports grants from GSK, DFG, EDCTP, BMBF, and Takeda outside the submitted work. All other authors report no potential conflicts. All authors have submitted the ICMJE Form for Disclosure of Potential Conflicts of Interest. Conflicts that the editors consider relevant to the content of the manuscript have been disclosed.
