# Supplementary figures and images for: First-in-human, Randomized, Double-blind Clinical Trial of Differentially Adjuvanted PAMVAC, A Vaccine Candidate to Prevent Pregnancy-associated Malaria
Source: Clin Infect Dis. 2019 Jan 10;69(9):1509–16. doi: 10.1093/cid/ciy1140 (PMC6792113; doi:10.1093/cid/ciy1140)

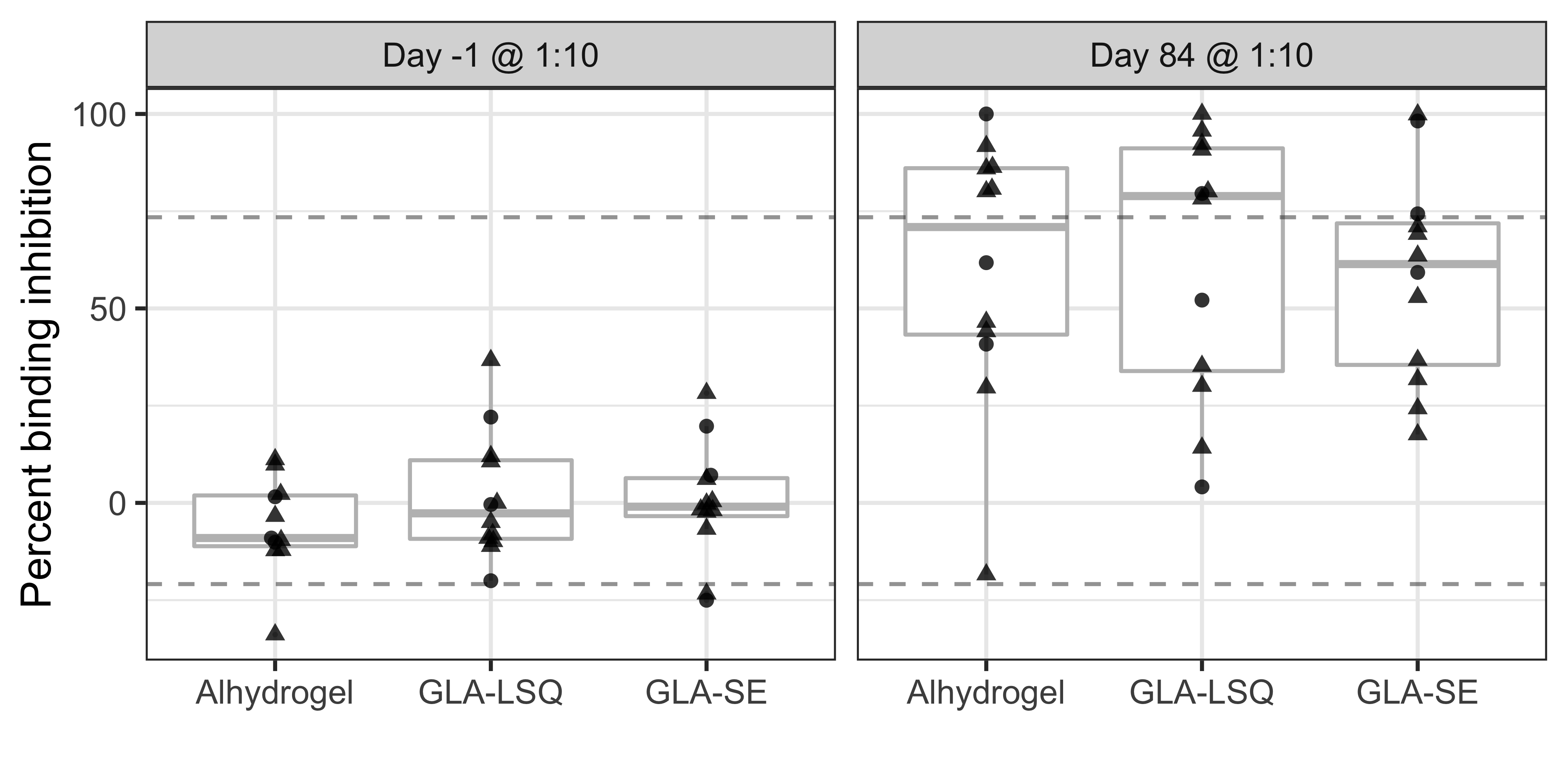

Supplement: ciy1140_suppl_Supplementary_Figure_S1 [file ciy1140_suppl_supplementary_figure_s1.png]

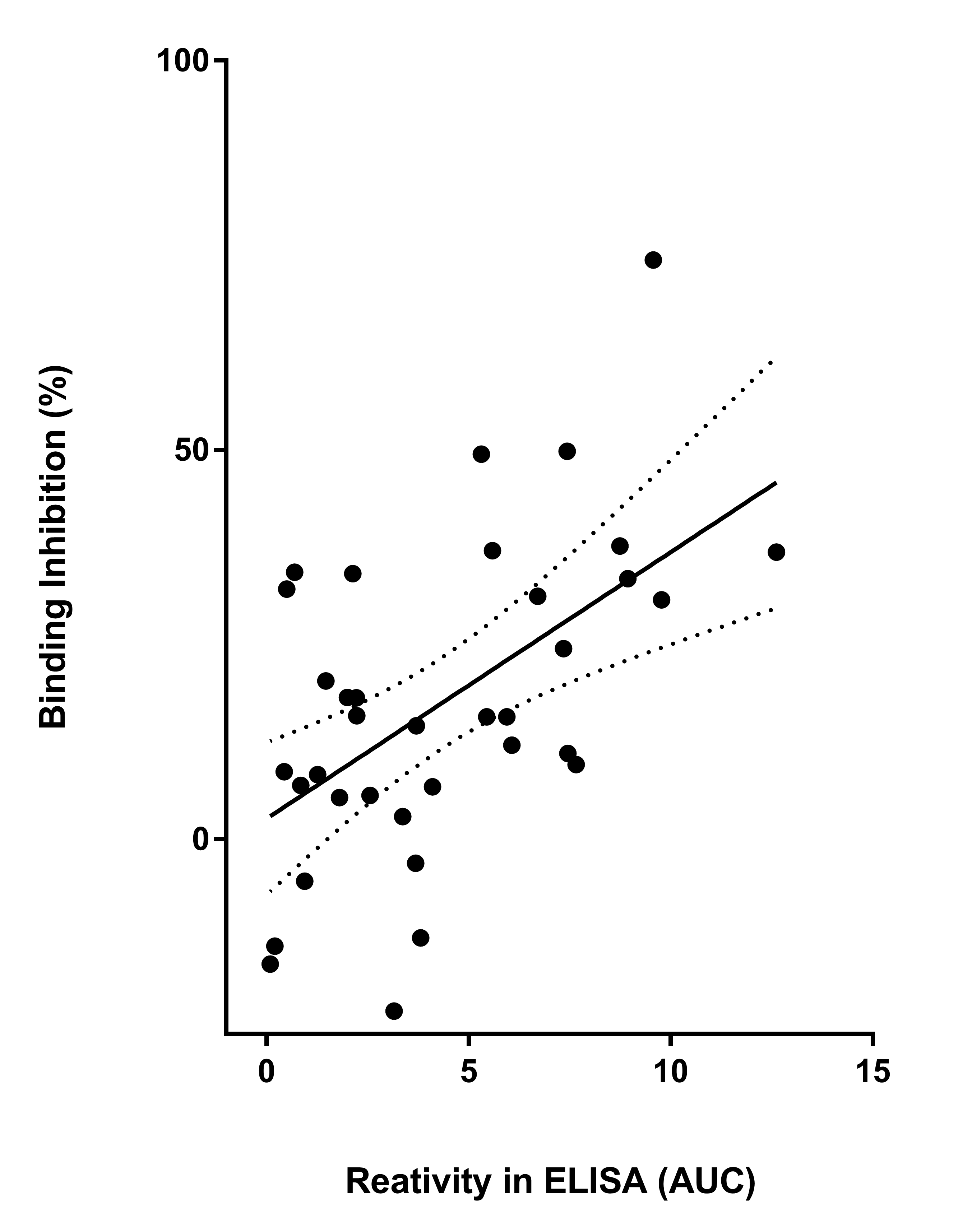

Supplement: ciy1140_suppl_Supplementary_Figure_S2 [file ciy1140_suppl_supplementary_figure_s2.png]

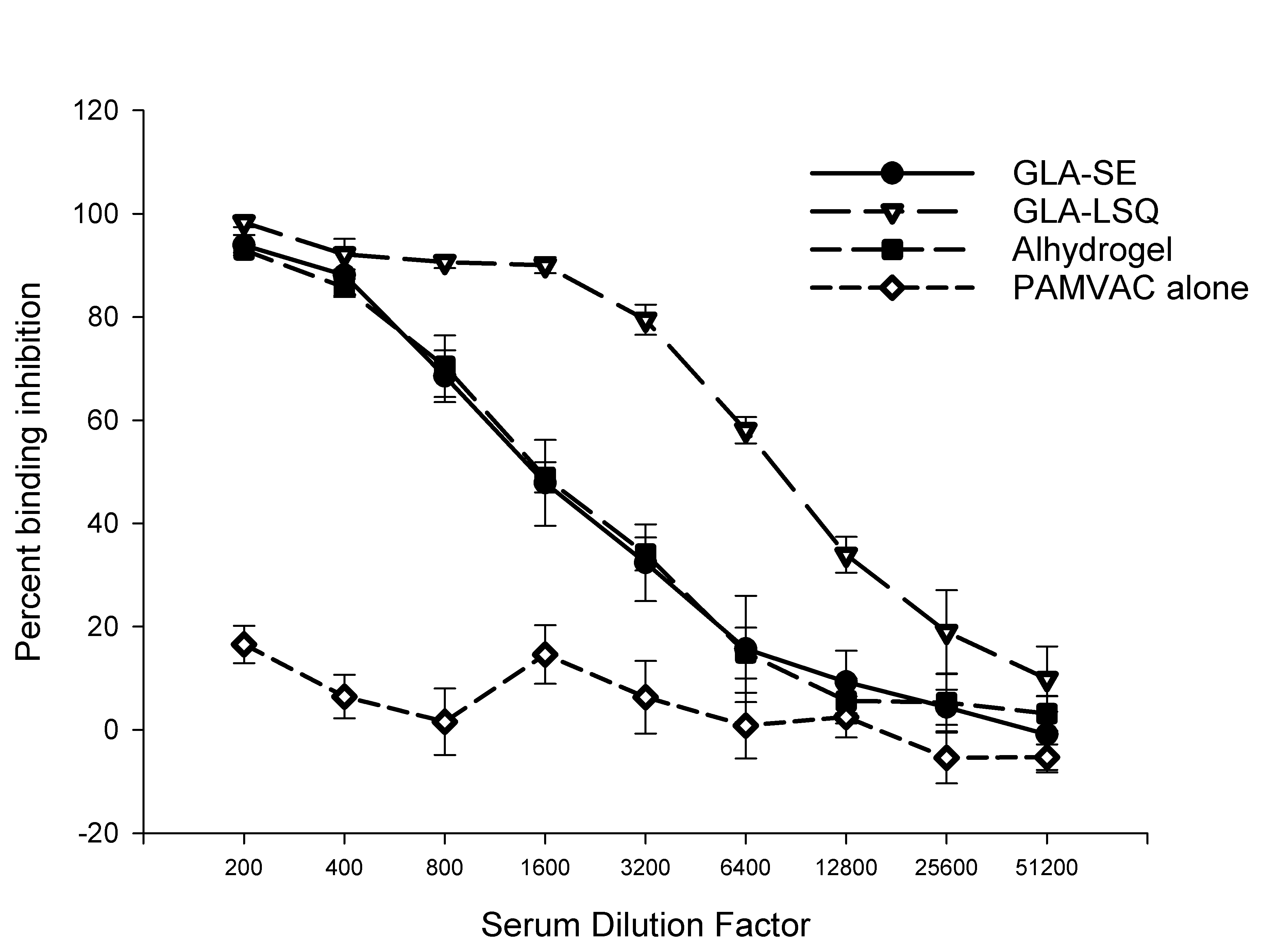

Supplement: ciy1140_suppl_Supplementary_Figure_S3 [file ciy1140_suppl_supplementary_figure_s3.png]
