# Supplementary material for: First-in-human, Randomized, Double-blind Clinical Trial of Differentially Adjuvanted PAMVAC, A Vaccine Candidate to Prevent Pregnancy-associated Malaria
Source: Clin Infect Dis. 2019 Jan 10;69(9):1509–16. doi: 10.1093/cid/ciy1140 (PMC6792113; doi:10.1093/cid/ciy1140)
Supplement: ciy1140_suppl_Supplementary_Table_1 [file ciy1140_suppl_supplementary_table_1.docx]

Supplementary Table 1: Adverse event pattern. Adverse Events were coded using MedDRA. The table shows MedDRA preferred terms of at least possibly related AE.

| **MedDRA high level group term** | **MedDRA preferred term** | **Severity** | **20/Al**  **(n = 3)** | **20/GLA-LSQ**  **(n = 3)** | **20/GLA-SE**  **(n = 3)** | **50/Al**  **(n = 9)** | **50/GLA-LSQ**  **(n = 9)** | **50/GLA-SE**  **(n = 9)** |
| --- | --- | --- | --- | --- | --- | --- | --- | --- |
| Administration site reactions | Injection site erythema | **1** | 0 | 1 (1) | 0 | 2 (2) | 4 (2) | 3 (2) |
|  | Injection site nodule | **1** | 1(1) | 1 (1) | 0 | 0 | 0 | 0 |
|  | Injection site pain | **1** | 2 (1) | 4 (2) | 3 (2) | 7 (6) | 9 (5) | 11 (6) |
|  |  | **2** | 0 | 0 | 0 | 1 (1) | 1 (1) | 3 (3) |
|  | Injection site paraesthesia | **1** | 0 | 0 | 0 | 1 (1) | 0 | 0 |
|  | Injection site pruritus | **1** | 0 | 0 | 1 (1) | 1 (1) | 4 (3) | 3 (3) |
|  |  | **2** | 0 | 0 | 0 | 0 | 1 (1) | 0 |
|  | Injection site swelling | **1** | 1 (1) | 1 (1) | 0 | 1 (1) | 1 (1) | 1 (1) |
|  |  | **2** | 0 | 0 | 0 | 0 | 0 | 2 (2) |
|  |  | **3** | 0 | 0 | 0 | 0 | 1 (1) | 0 |
| Body temperature conditions | Pyrexia | **1** | 0 | 0 | 0 | 0 | 1 (1) | 0 |
|  |  | **3** | 0 | 0 | 1 (1) | 0 | 0 | 0 |
| Cardiac disorder signs and symptoms | Palpitations | **1** | 1 (1) | 0 | 0 | 0 | 0 | 0 |
| Epidermal and dermal conditions | Rash vesicular | **1** | 0 | 0 | 0 | 0 | 1 (1) | 0 |
| Gastrointestinal signs and symptoms | Abdominal pain | **1** | 0 | 0 | 0 | 0 | 1 (1) | 0 |
| General system disorders NEC | Fatigue | **1** | 0 | 0 | 1 (1) | 1 (1) | 2 (2) | 0 |
|  | Flushing | **1** | 1 (1) | 0 | 0 | 0 | 0 | 0 |
| Headaches | Headache | **1** | 1 (1) | 0 | 2 (1) | 1 (1) | 0 | 0 |
|  |  | **2** | 1 (1) | 0 | 0 | 0 | 0 | 0 |
|  | Migraine | **2** | 0 | 0 | 0 | 0 | 0 | 1 (1) |
| Joint disorders | Arthralgia | **1** | 0 | 0 | 0 | 0 | 0 | 1 (1) |
| Metabolism disorders NEC | Hyperbilirubinaemia | **1** | 0 | 0 | 0 | 1 (1) | 0 | 0 |
| Muscle disorders | Myalgia | **1** | 1 (1) | 0 | 0 | 0 | 0 | 0 |
| Respiratory disorders NEC | Oropharyngeal pain | **1** | 0 | 0 | 0 | 1 (1) | 0 | 0 |
| Sleep disorders and disturbances | Hypersomnia | **1** | 0 | 0 | 0 | 0 | 1 (1) | 0 |
| Upper respiratory tract disorders (excl infections) | Nasal congestion | **1** | 0 | 0 | 0 | 1 (1) | 0 | 0 |

* number of AEs (number of volunteers)
