# Supplementary material for: First-in-human, Randomized, Double-blind Clinical Trial of Differentially Adjuvanted PAMVAC, A Vaccine Candidate to Prevent Pregnancy-associated Malaria
Source: Clin Infect Dis. 2019 Jan 10;69(9):1509–16. doi: 10.1093/cid/ciy1140 (PMC6792113; doi:10.1093/cid/ciy1140)
Supplement: ciy1140_suppl_Supplementary_Figure_Legends [file ciy1140_suppl_supplementary_figure_legends.docx]

Supplementary Figure 1. Functional activity of induced antibodies. The binding inhibitory activity of plasma against erythrocytes infected with VAR2CSA expressing FCR3 parasites binding to chondroitin sulfate A. The plasma was tested at Day 7 at a dilution of 1:10 and at Day 84 at a 1:10 dilution. Briefly, CSA was coated at the bottom of 96 well plates. Infected erythrocytes were added along with plasma. The plates were washed using a washing robot (Biomek 2000). Values shown are the percentage inhibition according to binding in wells without plasma after subtraction of background binding values in wells without CSA. All samples were run in triplicate. 50 µg group: dark blue; 20 µg group: light blue.

Supplementary Figure 2. Correlation between overall levels of IgG and functional activity. The graph shows area under the curves values as a measure of antibody levels against PAMVAC measured by ELISA and the level of binding inhibition at 1:20 dilution of plasma taken at day 84.

Supplementary Figure 3. Functional activity of induced antibodies in mice during pre-clinical development. The binding inhibitory activity of plasma against erythrocytes infected with VAR2CSA expressing FCR3 parasites binding to chondroitin sulfate A. The plasma tested was taken three weeks after third immunization as described [20]. Briefly, CSA was coated at the bottom of 96 well plates. Infected erythrocytes were added along with different dilutions of plasma. The plates were washed using a washing robot (Biomek 2000). Values shown are the percentage inhibition according to binding in wells without plasma after subtraction of background binding values in wells without CSA. All samples were run in triplicate.

Supplementary Figure 4. Sequence alignment of different antigens used to assess cross-reactivity in ELISA. The 3D7 and FCR3 *var2csa* variants belongs to two different clades due to the dimorphic region in DBL2 (AA 209-237 in the alignment). Reichenowi, Clin1 and Clin2 belong to the FCR3 clade in this block of variance. However, in other regions of variance FCR3 and 3D7 are more similar than the other sequences [40]. The WR80 clade after initiation of the clinical development is identified by a dimorphic region in ID1 [24]. Clin3 is the 3D7 sequence including DBL1 (not shown). The alignment was done in Clustal Omega Version 1.2.4 viewed in mView.
